# Study in 1790 Baltic men: *FSHR* Asn680Ser polymorphism affects total testes volume

**DOI:** 10.1111/j.2047-2927.2012.00028.x

**Published:** 2012-11-29

**Authors:** M Grigorova, M Punab, O Poolamets, S Sõber, V Vihljajev, B Žilaitienė, J Erenpreiss, V Matulevičius, I Tsarev, M Laan

**Affiliations:** * Human Molecular Genetics Research Group, Institute of Molecular and Cell Biology, University of TartuTartu, Estonia; † Andrology Unit, Tartu University ClinicsTartu, Estonia; ‡ Lithuanian University of Health Sciences, Medical Academy, Institute of EndocrinologyKaunas, Lithuania; § Andrology Laboratory, Riga Stradins UniversityRiga, Latvia

**Keywords:** FSH, FSH receptor, FSHR Asn680Ser, meta-analysis, testes volume

## Abstract

Follicle-stimulating hormone receptor (FSHR) contains two common linked polymorphisms, Thr307Ala (rs6165) and Asn680Ser (rs6166), shown to modulate ovarian function in women. The effect on male fertility and reproductive parameters has been inconclusive. We studied FSHR Asn680Ser polymorphism in a large study group (*n* = 1790) from the Baltic countries. The population-based Baltic male cohort (Estonians, Latvians, Lithuanians; *n* = 1052) and Estonian oligo-/azoospermic (sperm concentration <20 × 10^6^/mL) idiopathic infertile patients (*n* = 738) were genotyped for the FSHR Asn680Ser using PCR-RFLP. Genetic associations were tested using linear regression under additive model and results were combined in meta-analysis. No statistical difference was detected in allelic distribution of the FSHR Asn680Ser between the Baltic cohort and Estonian male infertility group. A consistent significant association was detected between the FSHR Ser680 allele and lower total testes volume in both, the Baltic cohort (*p =* 0.010, effect = −1.16 mL) and Estonian idiopathic infertility group (*p* = 0.007, effect = −1.77 mL). In meta-analysis, the statistical significance was enhanced (*p* = 0.000066, effect = −1.40 mL). Meta-analysis supported further associations with moderate effect between the FSHR Ser680 variant and higher serum FSH (*p* = 0.072), lower Inhibin B (*p* = 0.037) and total testosterone (*p* = 0.034). No statistically significant associations were identified with serum LH and estradiol, and sperm parameters. In conclusion, the study in 1790 Baltic men shows statistically highly significant association of the FSHR Asn680Ser with total testes volume and supportive association with serum reproductive hormone levels indicative to the functional effect of the alternative FSHR variants on male reproductive physiology.

## Introduction

Follicle-stimulating hormone (FSH) contributes profoundly to the regulation of human reproductive processes. In women, FSH binds to FSH receptor (FSHR) on the ovarian granulosa cells driving the hormonal and cellular events that control folliculogenesis and oocyte maturation (Edson *et al*., 2009). In men, FSHR is located on the testicular Sertoli cells that are critical for quantitatively and qualitatively normal spermatogenesis. Binding of FSH to FSHR activates proliferation of Sertoli cells in foetal, neonatal and pubertal development and promotes mitosis in spermatogonia (Nieschlag *et al*., 1999; Plant & Marshall, 2001; Ruwanpura *et al*., 2010). FSH receptor (FSHR) is coded by the *FSHR* gene (chr 2p21) consisting of 10 exons and encoding for 695 aa residues (Gromoll *et al*., 1996). *FSHR* exon 10 contains two common non-synonymous polymorphisms, Thr307Ala (c.919A > G; rs6165) and Asn680Ser (c.2139A > G; rs6166) that have been well characterized and extensively studied (Simoni *et al*., 1999). These two allelic variants are in near complete linkage disequilibrium and result in two discrete combinations of FSH receptor isoforms (Thr307-Asn680, Ala307-Ser680) (Simoni *et al*., 2002). FSHR isoforms are almost equally prevalent in studied European populations, whereas Asian and African populations exhibit slightly higher prevalence of the Thr307-Asn680 isoform (Kuijper *et al*., 2010; Lalioti, 2011). In women, Ala307-Ser680 variant has been associated with higher basal FSH levels during the follicular phase and a longer menstrual cycle (reviewed in Casarini *et al*., 2011; Lalioti, 2011). As the carriers of the FSHR Ala307-Ser680 isoform were consistently shown to require elevated FSH dosage in controlled ovarian hyperstimulation in in vitro fertilization (Perez *et al*., 2000; Behre *et al*., 2005), there is a potential for pharmacogenetic application in optimizing treatment (reviewed in Altmäe *et al*., 2011; Laan *et al*., 2012).

The effect of the two FSHR variants on male fertility and reproductive parameters has been inconclusive (Casarini *et al*., 2011; Lalioti, 2011; Laan *et al*., 2012). Majority of reports represent case-control studies comparing the distribution of alternative FSHR isoforms (Thr307-Asn680, Ala307-Ser680) in groups of infertile men vs. fertile controls. The carrier frequencies of the FSHR variants showed no significant difference between infertile and fertile men in individual studies performed in various ethnic groups (Simoni *et al*., 1999; Ahda *et al*., 2005; Pengo *et al*., 2006) and also in meta-analyses across several studies (Tüttelmann *et al*., 2007; Lend *et al*., 2010; Wu *et al*., 2012). The effect of FSHR isoforms on clinical reproductive parameters (e.g. FSH level, sperm count and total testes volume) has been tested in small patient groups below 350 individuals and has failed to show any consistent associations (reviewed in Lalioti, 2011). However, a fresh study on 313 Swedish young men cohort reported significantly lower serum FSH, but higher serum estradiol, sex hormone-binding globulin and total testosterone concentrations in Thr307-Asn680 homozygous men compared with heterozygotes and Ala307-Ser680 homozygotes (Lindgren *et al*., 2012). Thr307-Asn680 homozygotes had also larger testes volume and higher sperm counts. Previously, infertile men from Korea genotyped for Thr307Ala (Song *et al*., 2001) and from Egypt studied for Asn680Ser (Zalata *et al*., 2008) were shown to exhibit significantly higher serum FSH and lower total testes volume and serum testosterone in Ala307 or Ser680 variant carriers, respectively. There are limited data on the potential effect of the carrier status of alternative FSHR receptor isoforms on FSH treatment in male infertility (reviewed in Laan *et al*., 2012). A pilot pharmacogenetic study has shown that only the infertile male patients carrying Ser-allele of the FSHR Asn680Ser polymorphism showed significant increase in total sperm count, concentration, motility and percentage of normal morphology forms (Selice *et al*., 2011).

To overcome the sample-size limitations of previous studies and to reach conclusive evidence, we studied the effect of FSHR Asn680Ser polymorphism (rs6166) on male reproductive parameters in a large study group (*n* = 1790) involving population-based male cohort from three Baltic countries (*n* = 1052) and oligo-azoospermic (sperm concentration below 20 × 10^6^/mL) idiopathic infertile patients from Estonia (*n* = 738). Analysis in both study groups and in applying meta-analysis across samples showed small (G-allele effect −1.40 mL), but consistent and statistically highly significant (*p* = 0.000066) effect of the Ser680 variant on lower total testes volume. In addition, among idiopathic infertile patients, supported by meta-analysis results, trends for associations of downstream hormonal parameters (higher FSH, lower Inhibin B and total testosterone) were observed.

## Materials and methods

### Baltic male cohort

The Baltic male cohort was recruited between May 2003 and June 2004 among the participants in a prospective study Environment and Reproductive Health (EU 6th FP project QLRT-2001-02911) in parallel at the three study centres (Tartu, Estonia; Riga, Latvia; Kaunas, and Lithuania). Detailed principles of the study group formation have been described previously (Punab *et al*., 2002). The recruitment and phenotyping protocols of the study subjects at the participating centres were identical. Study participation was voluntary and written informed consent was obtained from all subjects. Men were recruited to the study at the Centre of Andrology, University Clinic of Tartu, Estonia (*n* = 578; all born and living in Estonia), at the Riga Family and Sexual Problems Centre, Latvia (*n* = 300; all born and living in Latvia) and at the specialized laboratory of the Institute of Endocrinology, Kaunas University of Medicine (*n* = 326; all born and living in Lithuania). In the current project, 152 subjects were excluded (lack of spermatozoa in ejaculate, i.e. azoospermia, *n* = 2; cryptorchidism, *n* = 13; abuse of anabolic steroids, *n* = 1; orchitis with unilateral testis damage, *n* = 1; incomplete data, *n* = 15; unavailable DNA sample, *n* = 120). The final number of analysed subjects was 1052 (Table 1). The study has been approved by the Ethics Committee of Human Research of the University Clinic of Tartu, Estonia (27.01.2003), the Ethics Committee of Riga Stradins University, Latvia (23.04.2003) and the Regional Ethics Committee of Kaunas, Lithuania (approval no. 13, 2003).

**Table 1:** General characteristics of the study groups^a^

General characteristics	Baltic male cohort (*n* = 1052)	Estonian idiopathic male infertility patients		
		
		Full study group (*n* = 738)	Oligozoospermic men (*n* = 684)^b^	Azoospermic men (*n* = 54)
Age (years)	20.1 ± 2.0 19.8 (17.4–24.0)	31.7 ± 6.1 31.0 (23.4–42.4)	31.7 ± 6.1 30.9 (23.4–42.4)	32.9 ± 5.8 32.6 (23.5–44.7)
BMI (kg/m^2^)	22.3 ± 2.6 22.1 (18.8–27.1)	26.7 ± 4.3 26.0 (21.0–34.7)	26.6 ± 4.4 25.9 (20.9–34.7)	27.4 ± 3.9 27.8 (21.3–35.8)
Abstinence period (hours)	108.4 ± 62.7 87.0 (49.0–232.2)	92.7 ± 50.1 72.0 (48.0–168.0)	92.0 ± 50.3 72.0 (48.0–168.0)	101.5 ± 47.0 96.0 (48.0–168.0)
Total testes volume (mL)	49.2 ± 10.2 50.0 (33.0–70.0)	39.6 ± 10.6 40.0 (22.0–56.0)	40.3 ± 10.1 40.0 (24.0–56.0)	30.8 ± 12.3 32.5 (8.3–50.0)
Sperm concentration (10^6^/mL)	81.4 ± 74.0 63.3 (9.0–214.3)	7.0 ± 6.0 5.4 (0.0–18.0)	7.6 ± 5.9 6.4 (0.1–18.0)	0.0 ± 0.0 0.0 (0.0–0.0)

a Data are presented as mean ± SD and median (5th–95th percentile).

b Study group of Estonian oligozoospermic patients consists of Estonian infertile patients after excluding infertile patients diagnosed with azoospermia (*n* = 54).

### Estonian idiopathic infertility patients

The details of the formation of the study group of Estonian idiopathic infertility patients are described elsewhere (Punab *et al*., 2003; Grigorova *et al*., 2010). In brief, the study group of Estonian men with idiopathic infertility (*n* = 750) was recruited at the Andrology Centre, Tartu University Clinics between June 2003 and August 2008 and consisted of male partners of couples failing to conceive a child for a period of ≥12 months. All study participants were of white European ancestry, born and living in Estonia. The inclusion criterion for male partners of infertile couples entering the study was sperm concentration below 20 × 10^6^/mL. All men with causal factors for male factor infertility (obstruction, cryptorchidism, chromosomal abnormalities, Y chromosome deletions, hypogonadotrophic hypogonadism, testicular diseases, sexual dysfunctions, androgen abuse, severe traumas and operation in genital area, chemo- and radiotherapy) were excluded from the analyses resulting in a study group of 738 men (age 31.7 ± 6.1 years) (Table 1). The analysed Estonian idiopathic infertility patients included 684 oligo- and 54 azoospermic men (Table 1). The study has been approved by the Ethics Committee of Human Research of the University Clinic of Tartu, Estonia (permissions no. 112/3, 27.01.2003; 117/9, 16.06.03).

### Hormone assays

For all participants of the study, venous blood was obtained from the cubital vein in the morning and serum was separated immediately. Serum sampling period for the Baltic cohort was from 08.00 to 13.00 (median 11.00), and for the Estonian infertility patients from 08.00 to 11.00 (median 9.30), respectively.

For the Baltic cohort, serum levels of FSH, LH and total testosterone were determined using time-resolved immunofluorometric assays (Delfia, Wallac, Turku, Finland), estradiol by radioimmunoassay (Pantex, Santa Monica, CA, USA) and Inhibin B by a specific two-sided enzyme immunometric assay (Serotec, Oxford, UK) at the Department of Growth and Reproduction in Copenhagen, Rigshospitalet, Denmark. The intra- and inter-assay coefficients of variation (CV) for measurement of both FSH and LH were 3 and 4.5%, for total testosterone <8% and <5%, for estradiol 7.5% and 13%, and for Inhibin B 15% and 18%, respectively. For the Estonian idiopathic infertility patients, the FSH, LH, total testosterone and estradiol levels of blood serum were measured using the Immulite automated chemiluminescence immunoassay analyser (Immulite; Diagnostic Products Corp., Los Angeles, CA) according to manufacturer's instructions. Inhibin B was determined in duplicate using a specific enzyme immunometric assay (Diagnostic Systems Laboratories, Inc., Webster, TX) at the United Laboratories, University of Tartu Clinics. The intra- and inter-assay CV were 4.2 and 8% for FSH; 4.0 and 7.1% for LH; 6.3 and 9.4% for testosterone; 7.5% and 13% for estradiol; 15% and 18% for Inhibin B.

### Semen analysis and physical examination

Semen samples were obtained by masturbation and all semen values were determined in accordance with the WHO criteria. In brief, after ejaculation, the semen was incubated at 37 °C for 30–40 min for liquefaction. Semen volume was estimated by weighing the collection tube with the semen sample and subsequently subtracting the predetermined weight of the empty tube assuming 1g = 1mL. For assessment of the sperm concentration, the samples were diluted in a solution of 0.6 mol/L NaHCO_3_ and 0.4% (v/v) formaldehyde in distilled water. The sperm concentration was assessed using the improved Neubauer haemocytometers.

Patients were examined by clinical investigators who had passed special training. Physical examination for the assessment of genital pathology and testicular size was performed with the man in standing position. If necessary, pathologies were clarified further with the men in supine position. The orchidometer (made of birch wood; Pharmacia & Upjohn, Copenhagen, Denmark) was used for the assessment of testicular size. The total testes volume is the sum of right and left testicles.

### Genotyping procedure

The *FSHR* position 2039 (Asn680Ser, rs6166 in dbSNP) was analysed as a restriction fragment length polymorphism (RLFP) as described previously (Sudo *et al*., 2002). Genomic DNA was extracted from peripheral blood using a modified version of the salting-out method (Aljanabi & Martinez, 1997). The genotyping of the alternative alleles (A/G) was conducted by PCR (forward/reverse primers: 5′-TTTGTGGTCATCTGTGGCTGC-3′/5′-CAAAGGCAAGGACTGAATTATCATT-3′) and restriction enzyme cutting (*BseNI*; Fermentas, Vilnius, Lithuania) approach. Standard electrophoresis in 2% agarose gel and 0.5 × Tris-borate EDTA buffer was used to separate the uncut PCR product (520 bp) representing the A allele (Asn) from the restricted PCR fragments (413 and 107 bp) corresponding to the G allele (Ser).

### Data analysis

For the descriptive statistics mean, standard deviation, median and 5–95th percentiles were calculated for general characteristics (age, BMI, ejaculation abstinence period) and main outcome variables (hormone, semen variables and total testes volume). Calculations of descriptive statistics were performed using PASW software Grad Pack 18.0 (spss Inc., Chicago, IL, USA).

Deviation from Hardy–Weinberg equilibrium (HWE) was assessed using exact test based on Markov Chain (MC) algorithm, implicated in the Genepop software (Version 4.0.10) (Rousset, 2008). Statistical difference between the two study groups in the distribution of *FSHR* rs6166 allele and genotype frequencies was assessed using Chi-squared test implicated in Genepop software using ‘population differentiation’ option (Rousset, 2008). The testing conditions were the following: dememorization = 10 000, batches = 1000, iterations = 10 000.

Genetic associations between the *FSHR* rs6166 SNP and male reproductive parameters were tested using PLINK version 1.07 (http://pngu.mgh.harvard.edu/purcell/plink/) (Purcell *et al*., 2007). If appropriate, the natural log transformation was used to obtain an approximate normal distribution of values. Marker-trait association testing was performed using multiple linear regression under additive genetic model. In Baltic male cohort, linear regression testing was performed with the adjustment for age, BMI, smoking status and recruitment centre. Hormone measurements were additionally corrected for blood sampling hour, and semen parameters were corrected for ejaculation abstinence period according to the analysis settings described previously (Grigorova *et al*., 2011). In case of Estonian idiopathic infertile patients, linear regression testing was performed with adjustment for age; semen parameters were additionally corrected for abstinence period. The stringent Bonferroni threshold for correction for multiple testing was estimated 0.05/16 = 3.13 × 10^−3^, taking into account the number of independent measurements (eight) and tested study samples (two). Results of individual study groups were combined in meta-analysis using the meta package (Schwarzer, 2010, http://CRAN.R-project.org/package=meta) for the statistical package r (http://www.r-project.org) using inverse variance method under fixed-effects model.

The prediction of the possible impact of FSHR Asn680Ser amino acid substitution on the molecular properties of the FSHR protein was performed using online programs PolyPhen (version 2) (http://genetics.bwh.harvard.edu/pph2/) (Adzhubei *et al*., 2010) and SIFT Human SNPs (http://sift.jcvi.org/) (Ng & Henikoff, 2001).

## Results

The Baltic male cohort (*n* = 1052; 20.1 ± 2.0 years; sperm concentration, 81.4 ± 74.0 mLn/mL; Table 1) and Estonian idiopathic infertile male patients (*n* = 738; 31.7 ± 6.1 years; sperm concentration, 7.0 ± 6.0 mLn/mL) were genotyped for the human *FSHR* polymorphism rs6166 (c.2139 A > G; Asn680Ser). No statistical difference was observed in *FSHR* rs6166 allele and genotype frequencies when comparing the Baltic cohort with Estonian infertility patients, as well as among the oligo- and azoospermia subgroups of infertile men (Fisher's exact test, *P*-value >0.70) (Table 2).

**Table 2:** Allele and genotype frequencies of the study groups stratified by *FSHR* rs6166 genotypes

		Allele frequencies^a^		Genotype frequencies^a^			
				
		A	G	A/A	A/G	G/G	HWE^b^
Baltic male cohort (*n* = 1052)	% n	60.0 (1265)	40.0 (843)	36.0 (379)	48.1 (507)	15.9 (168)	1.0
All Estonian idiopathic male infertility patients (*n* = 738)	% n	59.3 (884)	40.7 (606)	35.4 (264)	47.8 (356)	16.8 (125)	0.82
Oligozoospermic men (n = 684)	% n	59.5 (814)	40.5 (554)	35.8 (245)	47.4 (324)	16.8 (115)	0.69
Azoospermic men (*n* = 54)	% n	59.3 (64)	40.7 (44)	31.5 (17)	55.6 (30)	13.0 (7)	0.40

a Data presented as percentage with number of allele/genotype carriers in brackets; Chi-squared test for the distribution of *FSHR* rs6166 allele and genotype frequencies for pairs of individual study groups (e.g. Baltic male cohort vs. full study group of Estonian infertile patients, Estonian oligozoospermic patients vs. Estonian azoospermic patients, etc.), *p* > 0.70.

b HWE, test for Hardy–Weinberg equilibrium.

Statistically significant association was detected between the *FSHR* c.2139A > G G-allele (coding Ser680) and lower total testes volume in both the Baltic cohort (linear regression additive model, *p =* 0.010, G-allele effect = −1.16 mL) and Estonian idiopathic male infertility group (*p* = 0.007, effect = −1.77 mL) (Tables 3 and 4). The latter association was also significant when only oligozoospermia subgroup was analysed (*n* = 684; *p =* 0.011, G-allele effect = −1.41 mL; Table 4), and exhibited also a non-significant trend among the small group of azoospermic men (*n* = 54; *p <* 0.3, G-allele effect = −2.7 mL; Supporting Table 1). Mean total testes volume differed between AA (Asn680Asn) and GG (Ser680Ser) homozygote subgroups for 2.1 mL and 3.1 mL in the Baltic cohort and Estonian male infertile patient groups, respectively (Table 3). Meta-analysis across the Baltic cohort and Estonian male infertility group reached statistically highly significant association between the *FSHR* Ser680 variant and reduced total testes volume (*p* = 0.000066, resistant to Bonferroni correction for multiple testing; effect = −1.40 mL) (Table 4).

**Table 3:** Clinical parameters of the study samples stratified based on the *FSHR* rs6166 genotype of the participants

Parameter^a^	*FSHR* rs6166	Baltic male cohort (*n* = 1052)	All infertility patients (*n* = 738)^b^	Oligozoospermic patients (*n* = 684)^b^
FSH (IU/L)	A/A	3.0 ± 1.6	8.2 ± 8.5	7.1 ± 6.0
		2.6 (1.1–5.9)	5.6 (1.9–25.6)	5.4 (1.8–19.7)
	A/G	3.2 ± 1.7	8.5 ± 8.2	7.0 ± 5.6
		2.8 (1.1–6.6)	5.6 (1.8–25.2)	5.3 (1.7–18.8)
	G/G	3.1 ± 1.8	9.9 ± 7.8	9.1 ± 6.9
		2.7 (1.3–6.1)	7.0 (2.3–28.5)	6.8 (2.3–23.9)
LH (IU/L)	A/A	4.0 ± 1.6	4.7 ± 2.8	4.3 ± 2.0
		3.8 (1.8–6.8)	4.1 (1.7–9.6)	4.1 (1.7–8.2)
	A/G	4.0 ± 1.6	4.5 ± 2.4	4.2 ± 1.9
		3.8 (1.8–7.1)	3.9 (1.7–8.6)	3.7 (1.6–7.6)
	G/G	4.1 ± 1.8	4.8 ± 2.3	4.7 ± 2.1
		3.9 (1.8–7.6)	4.2 (2.0–9.2)	4.2 (2.0–8.6)
Inhibin B (pg/mL)	A/A	233.8 ± 75.8	90.6 ± 51.7	94.6 ± 50.6
		226.5 (121.9–381.2)	91.5 (10.1–180.3)	93.8 (10.6–182.9)
	A/G	223.1 ± 76.7	92.6 ± 66.8	98.2 ± 66.1
		212.0 (112.3–361.7)	76.7 (13.9–214.8)	81.0 (23.5–223.9)
	G/G	233.4 ± 86.6	73.8 ± 52.3	75.1 ± 52.8
		222.0 (109.2–397.6)	53.9 (10.0–175.1)	53.9 (10.0–175.2)
Total testosterone (nmol/L)	A/A	28.4 ± 8.6	19.2 ± 7.0	19.2 ± 7.0
		27.8 (14.9–44.7)	18.5 (9.8–32.0)	18.5 (9.8–31.0)
	A/G	27.1 ± 9.7	18.3 ± 6.4	18.3 ± 6.2
		25.8 (14.4–44.2)	17.7 (9.8–30.2)	17.7 (10.1–30.4)
	G/G	27.3 ± 8.8	18.1 ± 5.9	18.3 ± 5.9
		26.0 (15.2–45.4)	17.7 (10.4–28.6)	17.7 (10.3–28.8)
Estradiol (pmol/L)	A/A	96.2 ± 26.8	102.9 ± 42.3	103.0 ± 43.0
		92.0 (59.0–145.0)	87.7 (73.0–172.7)	86.5 (73.0–172.6)
	A/G	93.5 ± 25.4	99.4 ± 35.9	98.9 ± 33.6
		89.0 (60.0–140.7)	87.0 (73.0–159.3)	87.0 (73.0–157.0)
	G/G	93.3 ± 21.3	99.0 ± 30.4	99.5 ± 30.6
		91.0 (56.5–131.1)	89.7 (73.0–163.7)	90.3 (73.0–164.2)
Total testes volume (mL)	A/A	50.4 ± 10.5	40.7 ± 11.8	41.3 ± 11.7
		50.0 (33.0–70.0)	41.0 (23.0–59.9)	41.0 (24.0–60.0)
	A/G	48.5 ± 10.0	39.5 ± 9.8	40.3 ± 9.1
		50.0 (33.0–65.0)	40.0 (22.0–55.0)	40.0 (24.0–55.9)
	G/G	48.3 ± 10.1	37.6 ± 9.4	38.4 ± 8.9
		48.0 (32.0–70.0)	36.0 (22.1–53.8)	37.0 (23.7–54.0)
Semen volume (mL)	A/A	3.4 ± 1.6	4.4 ± 1.9	4.3 ± 1.9
		3.1 (1.3–6.4)	4.0 (1.9–8.1)	4.0 (1.8–8.1)
	A/G	3.5 ± 1.6	4.1 ± 1.8	4.2 ± 1.8
		3.4 (1.3–6.5)	3.9 (1.4–7.6)	4.0 (1.4–7.6)
	G/G	3.5 ± 1.6	4.0 ± 1.6	4.0 ± 1.6
		3.4 (1.1–6.5)	3.8 (1.4–7.2)	3.8 (1.6–7.2)
Sperm concentration (10^6^/mL)	A/A	80.4 ± 66.9	7.1 ± 5.8	7.6 ± 5.7
		64.8 (7.8–212.2)	6.2 (0.0–17.0)	7.0 (0.2–17.0)
	A/G	83.0 ± 81.2	6.9 ± 6.0	7.5 ± 5.9
		62.5 (9.0–223.7)	5.0 (0.0–18.0)	6.0 (0.1–18.0)
	G/G	78.4 ± 66.3	7.1 ± 6.5	7.6 ± 6.5
		62.3 (12.2–181.4)	5.1 (0.0–18.0)	5.6 (0.1–18.2)
Total sperm count (10^6^)	A/A	267.2 ± 251.6	31.4 ± 30.4	33.5 ± 30.3
		202.2 (18.8–718.9)	23.0 (0.0–89.1)	25.6 (0.5–91.6)
	A/G	280.4 ± 291.9	30.2 ± 31.9	33.0 ± 31.9
		210.9 (16.6–767.4)	18.0 (0.0–93.8)	22.7 (0.3–94.9)
	G/G	266.5 ± 278.0	29.0 ± 31.1	30.8 ± 31.2
		213.3 (26.0–671.2)	18.9 (0.0–95.7)	20.0 (0.4–95.9)

a Data are presented as mean ± SD and median (5–95^th^ percentile).

b Clinical parameters of azoospermic patients (*n* = 54) stratified based on the *FSHR* rs6166 genotype are presented in Supporting Table 1.

**Table 4:** Marker-trait association analysis results in individual study groups and results of meta-analyses

			Multiple linear regression testing in individual study groups			Meta-analysis
				
Parameter		Effect unit	Baltic cohort (*n* = 1052)	All idiopathic infertility patients (*n* = 738)^a^	Oligozoospermia subgroup (*n* = 684)^a^	Baltic cohort and infertility patients (*n* = 1790)
FSH	*p*-value b		0.201	**0.028**	**0.010**	0.072
	G-allele effect (SE) c	IU/L	0.08 (0.06)	0.58 (0.26)	0.59 (0.22)	0.11 (0.06)
LH	*p*-value		0.995	0.432	0.241	0.733
	G-allele effect (SE)	IU/L	0.00 (0.07)	0.07 (0.11)	0.12 (0.10)	0.02 (0.06)
Inhibin B	*p*-value		0.131	0.092	**0.046**	**0.037**
	G-allele effect (SE)	pg/mL	−5.11 (3.45)	−7.78 (5.07)	−9.12 (5.02)	−5.96 (2.85)
Total testosterone	*p*-value		0.084	0.174	0.428	**0.034**
	G-allele effect (SE)	nmol/L	−0.67 (0.40)	−0.46 (0.34)	−0.28 (0.35)	−0.55 (0.26)
Estradiol	*p*-value		0.176	0.431	0.560	0.124
	G-allele effect (SE)	pmol/L	−1.37 (1.03)	−1.24 (1.60)	−0.94 (1.64)	−1.33 (0.87)
Total testes volume	*p*-value		**0.010**	0.0017*	**0.011**	0.000066*
	G-allele effect (SE)	mL	−1.16 (0.45)	−1.77 (0.56)	−1.41 (0.55)	−1.40 (0.35)
Semen volume	*p*-value		0.953	**0.048**	0.156	0.993
	G-allele effect (SE)	mL	0.00 (0.07)	−0.18 (0.09)	−0.13 (0.10)	0.00 (0.07)
Sperm concentration	*p*-value		0.963	0.706	0.342	0.710
	G-allele effect (SE)	10^6^/mL	0.11 (2.45)	−0.11 (0.29)	−0.32 (0.37)	−0.11 (0.29)
Total sperm count	*p*-value		0.959	0.436	0.199	0.474
	G-allele effect (SE)	× 10^6^	0.43 (8.58)	−0.89 (1.22)	−1.72 (1.49)	−0.86 (1.21)

a Association testing among azoospermic patients (*n* = 54) is presented in Supporting Table 1.

b Significant associations (*p* < 0.05) are given in bold. Asterisk (*) marks *p*-values resistant to Bonferroni correction for multiple testing.

c rs6166 G-allele effect is shown as the estimated linear regression (additive model) statistic β, standard error of the regression (SE) is shown in brackets.

Among the studied Estonian idiopathic infertility group, the *FSHR* Ser680 variant (G-allele) showed also significant or suggestive association with higher serum FSH level (*p* = 0.028, effect = 0.58 IU/L), lower Inhibin B (*p* = 0.092, effect = −7.78 pg/mL) and reduced semen volume (*p* = 0.048, effect = −0.18 mL) (Tables 3 and 4). The association with higher circulating FSH (*p* = 0.01) and lower Inhibin B (*p* = 0.046) was also significant in the oligozoospermia subgroup. Interestingly, meta-analysis across the Estonian idiopathic infertility group and the Baltic male cohort reached statistically significant association not only with lower serum Inhibin B (*p* = 0.037, effect = −5.96 pg/mL) but also with reduced total testosterone level (*p* = 0.034, effect = −0.55 nmol/L) (Table 4). Meta-analysis did not support the association with semen volume.

No statistically significant associations were identified with serum LH and estradiol, and sperm parameters (sperm concentration, total sperm count) either in individual studies or in meta-analysis (Tables 3 and 4).

## Discussion

There has been a shortage of conclusive evidence on the association of the two common FSHR isoforms (Thr307-Asn680, Ala307-Ser680) (Simoni *et al*., 2002) with male reproductive parameters. The present analysis of *FSHR* Asn680Ser polymorphism (rs6166) in 1790 Baltic men represents the largest conducted study addressing this issue. No difference was observed in the allele frequencies of *FSHR* Asn680Ser polymorphism between population-based Baltic control cohort and Estonian idiopathic infertility patient group. This supports a recent meta-analysis of over 1644 infertile patients and 1748 fertile controls indicating lack of association with male infertility (Wu *et al*., 2012).

We report conclusive data on the association of the FSHR Ser680 (G-allele) variant with total testes volume (*p* = 0.000066, G-allele effect −1.40 mL). This association is consistent with the published data in Swedes (Lindgren *et al*., 2012), Egyptians (Zalata *et al*., 2008) and Koreans (Song *et al*., 2001), and is independent whether the analysis was performed on fertile or infertile men. Previously, majority of studies have failed to detect any link between FSHR common isoforms and quantitative male reproductive parameters. The limitations of these studies might have been either small study groups with insufficient power to detect small phenotypic effects, analysed patients with severely disturbed reproductive physiology and hormonal feedback mechanisms and/or non-adjustment of the analysis for population-specific allele frequencies in multinational studies (Kuijper *et al*., 2010). Thus, meta-analysis across individual studies is recommended instead of increasing the statistical power by pooling the individual study groups. An additional uncertainty may be introduced by pooling individual studies owing to marked variability in performance of automated commercial immunoassays (Sikaris *et al*., 2005).

It is noteworthy that the identified effect of the *FSHR* Asn680Ser polymorphism on testicular volume is manifold smaller compared with the recently discovered *FSHB* promoter variant -211G/T (rs10835638; T-allele frequency in the Baltic cohort 12.9% (Grigorova *et al*., 2011). The *FSHB* -211G/T is a major gene variant affecting serum FSH concentrations in men (Grigorova *et al*., 2008, 2010, 2011). The median serum FSH in TT- compared with GG- homozygotes was 78% in the Baltic cohort and 48.5% in Estonian idiopathic infertile patient group. The total testes volume in *FSHB* -211 TT- compared with GG-genotype carriers was reported in average ∼9 mL (20%) smaller in the Baltic young men and ∼5.3 mL (13%) lower in the Estonian idiopathic infertility patients (Grigorova *et al*., 2010, 2011). In comparison, in this study, the *FSHR* Ser680Ser homozygotes have in average 2.1–3.1 mL (4–7%) smaller testes compared with Asn680Asn genotype carriers. The outcome of genetic association studies of both polymorphisms indicates that normal testes development requires adequate FSH stimulation of Sertoli cells during male foetal, early neonatal and pubertal development. In rats, suppression of neonatal FSH concentration was shown to reduce the final number of Sertoli cells by about 40% (Atanassova *et al*., 1999).

Several clinical studies have demonstrated the impact of the *FSHR* Asn680Ser polymorphism on FSH action in female in vivo (reviewed in Casarini *et al*., 2011). The current study along with previous reports convincingly supported the association of *FSHR* Ser-allele with lower total testes volume in men. However, so far there is limited in vitro data on the molecular basis of the alternative FSHR isoforms to explain the observed phenotypic effects in human. ‘In silico’ analysis using PolyPhen2 (Adzhubei *et al*., 2010) and SIFT Human Coding SNPs (Ng & Henikoff, 2001) predicts *FSHR* Asn680Ser substitution as ‘benign’ and ‘tolerated’, that is, with no major effect on protein function. A recent in vitro study evaluated the cellular mechanisms of FSHR Asn680Ser substitution in human granulosa cells (GC) retrieved from patients homozygous for the FSHR SNP (Asn/Asn; Ser/Ser) and undergoing ovarian stimulation (Nordhoff *et al*., 2011). Basal oestradiol, but not progesterone, concentrations on day 1 of GC culture, were significantly higher in Asn/Asn compared with Ser/Ser (*p* = 0.045), but non-responsive to FSH stimulation. The authors conclude from their experimental series that factors downstream of progesterone production or external to GC might be involved in the clinically observed differences in an FSHR variant-mediated response to FSH in women. Additional line of evidence is provided by a male clinical study based on the hypothesis that men with at least one Serine in FSHR position 680 would have lower sensitivity to FSH and their FSH basal levels are not sufficient for optimal stimulation of spermatogenesis (Selice *et al*., 2011). Consistent with their hypothesis, the authors showed that standard FSH treatment improved spermatogenesis in subjects carrying FSHR Ser680 allele, which was not the case for the FSHR Asn680Asn genotype carriers. This observation was explained that in the Asn680Asn subjects the same FSH basal levels are already operating at their maximal potential on stimulation of spermatogenesis (Atanassova *et al*., 1999).

In addition, the current study identified in the *FSHR* Ser680 male carriers moderately increased serum FSH and decreased Inhibin B and testosterone levels, which indirectly may indicate reduced Sertoli cell numbers. To our knowledge, this is the first study to analyse the association of FSHR Asn680Ser with serum Inhibin B concentrations and we demonstrate moderate, but consistent reduction in Inhibin B levels in carriers of FSHR Ser680 (meta-analysis: G-allele effect = −5.96 pg/mL; *p* = 0.037). Higher male serum FSH and lower total testosterone in FSHR Ala307-Ser680 carriers are consistent with previous reports in European and Asian populations (Song *et al*., 2001; Zalata *et al*., 2008; Lindgren *et al*., 2012). However, our study (*n* = 1790) could not confirm statistically significant effect of the carrier status of the *FSHR* isoforms on serum estradiol and sperm parameters shown in a study on Swedish young men (*n* = 313; Lindgren *et al*., 2012). The observation of increased serum FSH levels in FSHR Ser680 carriers is also in line with reported data on higher circulating FSH in female FSHR Ala307-Ser680 variant carriers explained by a lower sensitivity of this FSHR isoform to bind FSH in women (reviewed in Laan *et al*., 2012).

In the current study, the effect of the FSHR Ser680 allele appeared to be stronger among idiopathic infertility patients compared with young men. The possible explanation may rely on physiological differences between Baltic male cohort (age 20.1 ± 2.0 years) and infertile male study group (age 31.7 ± 6.1 years). In the latter sample, the effect of *FSHR* Asn680Ser may become more pronounced because of the age-related decline in male fertility and accumulation of various fertility problems causing reduced fertility potential (Kühnert & Nieschlag, 2004; Harris *et al*., 2011). Among the participants of the Baltic male cohort of young men compensatory mechanisms may be involved acting to maintain maximum fertility potential.

In summary, the study in 1790 Baltic men from two independent studies shows convincing association of FSHR Asn680Ser polymorphism with total testes volume indicative to the functional effect of the carrier status of alternative *FSHR* gene variants on male reproductive physiology.

## SUPPORTING INFORMATION

**Table S1.** Distribution of reproductive parameters of idiopathic infertile male patients with azoospermia (*n* = 54) stratified by *FSHR* rs6166 genotype, shown as mean ± SD and median (5–95th percentiles)
