# Supplementary material for: Study in 1790 Baltic men: FSHR Asn680Ser polymorphism affects total testes volume
Source: Andrology. 2012 Nov 29;1(2):293–300. doi: 10.1111/j.2047-2927.2012.00028.x (PMC3674532; doi:10.1111/j.2047-2927.2012.00028.x)
Supplement: Supplementary file 1 [file andr0001-0293-SD1.docx]

**Supporting Table 1.** Distribution of reproductive parameters of idiopathic infertile male patients with azoospermia (n=54) stratified by *FSHR* rs6166 genotype, shown as mean ± SD and median (5-95th percentiles).

|  | ***FSHR* rs6166** | | | **multiple linear regression testing** |
| --- | --- | --- | --- | --- |
| **Parameter** | **A/A**  **(n=17)** | **A/G**  **(n=30)** | **G/G**  **(n=7)** | ***P*-value**  **G-allele effect (SE)** |
| **FSH (IU/L)** | 24.5 ± 18.1  18.1 (4.7 – 63.0) | 25.1 ± 12.9  22.9 (7.6 – 61.5) | 22.8 ± 11.4  28.4 (8.4 – 38.6) | 0.951  -0.17 (2.87) |
| **LH (IU/L)** | 9.2 ± 6.2  6.6 (1.6 – 25.1) | 7.9 ± 4.5  6.0 (3.6 – 19.4) | 6.8 ± 4.2  6.7 (1.8 – 12.2) | 0.407  -0.73 (0.98) |
| **Inhibin B (pg/mL)^a^** | 26.3 ± 11.7  28.0 (10.0 – 43.6) | 22.9 ± 19.5  19.6 (0.7 – 59.0) | 48.6 ± 44.4  48.6 (17.2 – 80.0) | 0.953  -0.47 (9.80) |
| **Total testosterone (nmol/L)** | 19.0 ± 7.3  18.1 (8.2 – 34.1) | 17.7 ± 8.3  17.1 (4.4 – 36.2) | 13.3 ± 2.8  11.9 (11.6 – 18.2) | 0.485  -1.14 (1.76) |
| **Estradiol (pmol/L)** | 102.6 ± 32.6  97.6 (73.0 – 180.0) | 105.3 ± 57.9  83.3 (73.0 – 302.0) | 89.3 ± 28.4  74.8 (73.0 – 139.0) | 0.373  -6.53 (7.83) |
| **Total testes volume (mL)** | 33.5 ± 12.0  34.0 (6.0 – 50.0) | 30.7 ± 13.1  33.0 (7.7 – 49.5) | 24.9 ± 8.1  26.0 (13.0 – 34.0) | 0.299  -2.72 (2.59) |
| **Semen volume (mL)** | 4.7 ± 2.2  3.8 (1.9 – 8.3) | 3.7 ± 1.7  3.4 (1.0 – 6.6) | 3.1 ± 1.9  2.6 (0.5 – 5.8) | 0.101  -0.61 (0.43) |

^a^ Inhibin B values are available for 19 individuals (AA, n=6; AG, n=11; GG, n=2).
